# Efficacy of Non-Surgical Periodontal Therapy with Adjunctive Methylene Blue and Toluidine Blue O Mediated Photodynamic in Treatment of Periodontitis: A Randomized Clinical Trial

**DOI:** 10.3390/clinpract14030076

**Published:** 2024-05-22

**Authors:** Kashan Kamal Najm, Sarhang Sarwat Gul, Ali Abbas Abdulkareem

**Affiliations:** 1 Department of Periodontics, College of Dentistry, University of Sulaimani, Sulaymaniyah 46001, Iraq; kashan.najm@univsul.edu.iq; 2 Medical Laboratory Department, College of Health and Medical Technology, Sulaimani Polytechnic University, Sulaymaniyah 46001, Iraq; 3 Department of Periodontics, College of Dentistry, University of Baghdad, Baghdad 10011, Iraq; ali.abbas@codental.uobaghdad.edu.iq

**Keywords:** periodontitis, periodontal therapy, photodynamic therapy, methylene blue, toluidine blue O

## Abstract

Background: This study aimed to examine the efficacy of methylene blue (MB) and toluidine blue O (TBO) photodynamic therapy (PDT) as adjuncts to root surface debridement (RSD). Methods: This split-mouth, randomized, controlled clinical trial included eighteen patients, and a total of 332 sites (control = 102, MB = 124 and TBO = 106) were examined. Two sessions of PDT were completed at baseline and two weeks after RSD. Clinical parameters of bleeding on probing (BOP), plaque index (PI), probing pocket depth (PPD), and clinical attachment level (CAL) were measured pre- and post-treatment. Results: PPD and BOP reductions in sites treated by RSD with adjunctive photosensitizers (MB and TBO) were significantly higher than in control sites. RSD with MB showed higher efficacy in improving moderately deep pockets (OR 3.350), while adjunctive TBO showed better results in treating deeper pockets (OR 4.643). Conclusions: Results suggested that adjunctive use of MB and TBO to RSD could significantly improve periodontal pocket closure and reduce signs of inflammation. In addition, TBO seems to be more efficient in treating deep periodontal pockets than MB, which is more effective in resolving shallower pockets.

## 1. Introduction

Dental biofilm is the main factor responsible for triggering inflammatory events leading to the development of periodontal diseases (PD). Dental biofilm initially accumulates supragingivally, then progresses to subgingival niches [1]. The dysbiotic bacterial biofilm and the associated virulence factors, such as lipopolysaccharides, induce the recruitment of leucocytes, neutrophils, and T lymphocytes, as well as the secretion of antibodies, and chemical inflammatory cytokines and chemokines [2,3]. These inflammatory events lead to the development of periodontitis, which is responsible for irreversible damage to periodontal tissues that finally results in tooth loss [4].

Periodontal treatment basically aims to eradicate supra- and subgingival biofilm masses, minimizing the pathogenic bacteria load responsible for the progression of PD [5]. Non-surgical periodontal treatment, root surface debridement (RSD), is used to remove dental biofilm and eradicate pathogenic organisms, with various degrees of clinical success [6]. RSD is the core of phase 1 periodontal therapy, aiming to mechanically remove established biofilms from the subgingival microenvironment [7]. However, bacteria may shelter in areas not accessible to curettes or ultrasonic devices by deeply penetrating the cementum and dentinal tubules or other reservoirs inside the oral cavity to periodontal sites [2]. Additionally, certain anatomical sites, including the most apical extent of deep periodontal pockets, root surface concavities, or complex architecture of the furcation area, can also compromise the efficacy of RSD [8]. Locally or systemically delivered antimicrobials have been constantly proposed as adjuncts to RSD to solve these issues. These adjunctive treatments have shown a good potential to minimize the load of periodontal pathogens in sites normally not easily cleaned by RSD [9]. However, skepticism is always associated with the use of systemic antibiotics due to side effects and the development of bacterial resistance [10]. Therefore, interest in finding an alternative antimicrobial drug has increased.

Photodynamic therapy (PDT) is a promising substitute for antibiotics which is principally reliant on the photosensitization of bacteria with exogenous compounds called photosensitizers (PS) [11]. The latter agent absorbs light in the presence of molecular oxygen, releasing free radicals together with singlet oxygen which are cytotoxic to microorganisms [12]. Several systematic reviews evaluated the efficacy of PDT as adjunct to periodontal therapy [13,14,15]. These reviews covered the application of different PS such as methylene blue (MB), toluidine blue O (TBO), and indocyanine green-mediated using different sources of light activation. The results contrasted between significant and partial improvement in clinical and microbiological outcomes as compared to RSD alone. However, all these studies agreed on the heterogeneity of the results and the need for conducting further clinical trials to confirm these findings.

PDT has been proposed as a novel tool as adjunct to RSD to treat PD, which has shown promising results in improving the clinical periodontal parameters of plaque index (PI), bleeding on probing (BOP), clinical attachment level (CAL), and probing pocket depth (PPD) [16]. However, evidence from randomized clinical trials has shown conflicting outcomes regarding the improvement in clinical periodontal parameters and periodontal pathogen elimination when PDT is used in conjunction with RSD [17,18]. The purpose of this study was to evaluate the efficacy of the photosensitizers, MB and TBO, as adjuncts to RSD in treatment of periodontitis.

## 2. Materials and Methods

### 2.1. Study Design, Settings, and Recruitment

This pilot trial followed a split-mouth design in which periodontal pockets were randomly assigned as controls or receiving adjunctive therapy (MB or TBO). Eighteen participants (nine males and nine females) were recruited from the Department of Periodontics, College of Dentistry, University of Sulaimani. Patients who were diagnosed with periodontitis and exhibited PPD ≥ 5 mm were enrolled from December 2021 to January 2023.

The study followed the Declaration of Helsinki (amended in Edinburgh, 2000) in regard to human research and was approved by the Ethics Committee of College of Dentistry of the University of Sulaimani (ethical approval number: 55/21 on 11 March 2021). The clinical trial was registered at the Clinical Trial Registry of the US National Library of Medicine (NIH) (Clinicaltrial.gov, access date: 21 May 2024) with registration no. NCT05162417 under the protocol record title (University of Sulaimani Protocol Record 55/21, Photodynamic in Periodontal Treatment). Informed consent, including the use of their photographs, was obtained from each patient before participation in the study.

### 2.2. Sample Size Calculation

The sample size was calculated based on the reduction of PPD after 3 months following termination of active periodontal therapy, considering a 5% alpha error [19]. Therefore, with a sample size of 14 patients, the power of the study was calculated to be 80%. An effect size of 0.9 and α = 0.05 were obtained using G-Power analysis software (G*Power 3.1.9.7, Franz Faul, Universitat Kiel, Keil, Germany). To account for any possible drop out during the trial, the sample size was increased by 25%, which was rounded to 20 patients.

### 2.3. Inclusion and Exclusion Criteria

Patients with periodontitis were defined by the presence of CAL interdentally at two or more non-adjacent teeth or CAL affecting buccal/lingual aspects associated with PPD > 3 mm in ≥2 teeth [20]. The prerequisite for participation in the study was at least three non-adjacent teeth with unstable periodontal pockets (PPD ≥ 4 mm with BOP) [20], and teeth with furcation involvement were not included. The exclusion criteria were smokers, patients with a history of systemic diseases such as diabetes mellitus and cardiovascular disease, and consumption of medications with an inhibitory or promoting effect on periodontal healing, including anticoagulants, anti-inflammatories, and antibiotics, within the last six months. Additionally, pregnant or nursing women and patients who had received periodontal therapy within the previous six months or were allergic to the test product were excluded [21].

### 2.4. Periodontal Parameters

The periodontal parameters of PI, BOP, CAL and PPD were measured at six sites per tooth (mesio-buccal, mid-buccal, disto-buccal, mesio-lingual, mid-lingual, and disto-lingual). The PI and BOP were assessed at baseline and 1-month and 3-month timepoints, whereas CAL and PPD were assessed at baseline and 3-month timepoints. The PI and BOP were recorded as present or absent. All measurements were performed by the same calibrated examiner using UNC 15 periodontal probe (UNC 15, Hu-Friedy, Chicago, IL, USA) at baseline and subsequent visits to ensure blindness [22]. Additionally, the statistician was also blinded from the details of each group. Before conducting the study, intraexaminer calibration of the examiner was approved when the interclass coefficient was >70% for continuous variables and the kappa coefficient > 90% for categorical variables. Periodontal parameters were recorded from five volunteers, and calibration sessions were repeated until the desired level of consistency was achieved.

### 2.5. Randomization and Treatment Protocol

For each patient, the mouth was divided into quadrants, each one having to contain moderate (4–6 mm) and deep (>6 mm) pockets. These quadrants were randomly assigned by lottery, using envelopes, into the control (C) group which received RSD alone and two test groups which received adjunctive MB- or TBO-mediated PDT.

**Figure 1:**
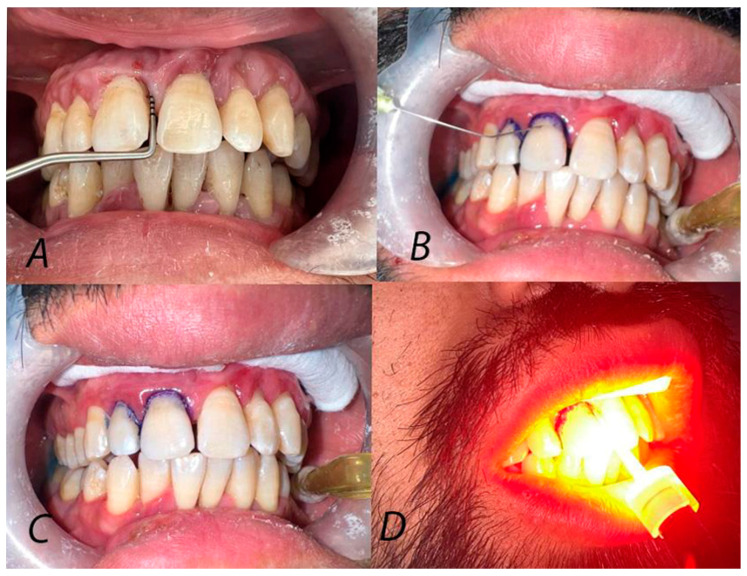
Steps of applying PDT: (**A**) Probing of test sites at the baseline. (**B**) Dye injection into the periodontal pockets. (**C**) Irrigation of the dye at the test sites. (**D**) Irradiation with light.

One calibrated clinician (KKN), other than the clinician who performed the measurements, provided the treatment, starting with an ultrasonic scaler followed by the application of adjunctive MB and TBO for test groups. The teeth to be treated with PS were isolated with cotton rolls to provide dryness at the treatment sites. A fresh stock solution, no more than 48 h old, of 1 mg/mL concentration was prepared for both MB and TBO (Biochem, Cosne-Course-Sur-Loire, France) and stored at 4 °C. A double-vented blunt endodontic irrigating needle (gauge 27) was inserted gently in the periodontal pocket to deliver MB and TBO. The PS were allowed to sit in the pocket for two minutes before the light irradiation, to allow the material absorption by bacteria. The excess PS from the pocket was flushed with 0.9% normal saline solution, and the sites were dried once again. Each site was illuminated with a 635 nm red LED source for one minute with a total light dose of 120 J/cm^2^, as shown in Figure 1. A Fiber-optic tip (200 μm diameter) was fixed on the light source to facilitate light transmission into the deep pockets, and the tip was held very close to the diseased site and positioned perpendicular to the surface of the gingival mucosa. To avoid the cross effect, sites treated with PS were in different quadrants, and the treatments were not conducted simultaneously, i.e., the application and activation of the first PS was finished before starting the next one.

After treatment, all patients were advised to perform adequate oral hygiene measures (both in test and control sites). These included teeth brushing three times a day and daily use of dental floss. After two weeks, the patients were recalled for the second PDT application. The BOP and PI were measured at a 1-month time point, while all clinical parameters (PI, BoP, PPD, and CAL) were re-measured 3 months after the first session of treatment.

### 2.6. Primary and Secondary Outcomes

The primary outcome was the mean reduction in PPD for moderate (4–6 mm) and deep (>6 mm) pockets by 1.29 mm and 2.16 mm, respectively, after 3 months of non-surgical therapy as previously reported [23]. The secondary outcomes were improvements in the other periodontal clinical parameters: PI, BOP, and CAL.

### 2.7. Statistical Analysis

The normality test, Shapiro–Wilk, was used first to confirm if the data were normally distributed or not. Accordingly, inferential intragroup comparisons for PPD at baseline and after 3 months were performed by Wilcoxon’s signed-rank test. The effect size of both PS was calculated by using Cohen’s d formula. To compare PPD for all groups, the mean differences between the baseline and endpoint (3 months) were calculated and compared by Kruskal–Wallis test followed by a post hoc test. Frequencies of moderate and deep periodontal pockets at baseline and endpoint were compared by a Chi-square test and odds ratio. For CAL, data were normally distributed; therefore, a paired *t*-test was used for pre- and post-treatment data of the same group and an ANOVA test for multi-group comparisons, along with Tukey’s post hoc test when needed. The latter was also used for intergroup analyses of PI and BOP which were normally distributed. While intragroup comparisons of these two indices at three time points were performed by Friedman test. GraphPad Prism software, version 8.4.0 for Win 10 (GraphPad Software Inc., San Diego, CA, USA) was used for statistical analyses. The statistical significance was defined as *p* ≤ 0.05.

## 3. Results

### 3.1. Study Population

Overall, 2 out of 20patients did not comply with the scheduled appointments, leaving 18 participants (9 males and 9 females) for final analysis. Their ages ranged between 23 and 63 years (43.44 ± 10.87). In total, 332 sites were examined and divided into control (*n* = 102), MB (*n* =124), and TBO (*n* =106) (Table 1). No postoperative complications were observed during the whole study period.

### 3.2. Primary Outcome

The reduction in PPD 3 months after RSD was considered as the primary outcome measure for this study. Intragroup comparisons indicated a significant reduction in PDD in all study groups, but with moderate (0.5) to high (0.7) effect size for TBO and MB, respectively (Table 2). Intergroup comparisons showed that pocket reduction in sites treated by RSD with adjunctive photosensitizers (MB and TBO) was significantly higher than in control sites (Table 2). Further analysis, based on the depth of the pockets, showed a similar pattern, whether a moderate (4–6 mm) or deep (>6 mm) pocket, when treated with MB and TBO (Table 2).

The percentage of improved sites, achieving mean PPD ≥ 1.29 mm for moderate pockets and ≥2.16 mm for deep pockets, was significantly higher in association with photosensitizers (Table 3). The chances of pocket closure improved with adjunctive use of MB (OR 3.422) and TBO (OR 2.772) as compared to RSD only. Interestingly, RSD used with MB showed higher efficacy in improving moderate PPD (OR 3.350), while adjunctive TBO showed better results in treating deep PPD (OR 4.643) (Table 3).

### 3.3. Secondary Outcomes

**Figure 2:**
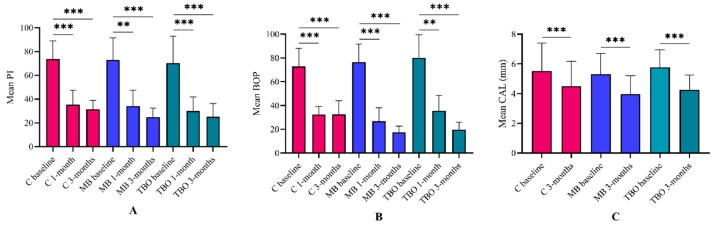
Intragroup comparisons of (**A**) plaque index (PI), (**B**) bleeding on probing (BOP), and (**C**) clinical attachment level (CAL). All treatments significantly improved periodontal parameters. ** *p* < 0.002, *** *p* < 0.001 using paired *t*-tests.

**Figure 3:**
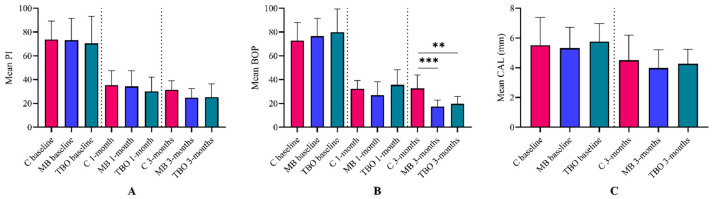
Intergroup comparisons of (**A**) plaque index (PI), (**B**) bleeding on probing (BOP), and (**C**) clinical attachment level (CAL). No significant differences in periodontal parameters were observed between sites treated with adjunctive photosensitizers and controls. The only exception was BOP after 3 months, when controls exhibited significantly higher scores than MB and TBO. ** *p* < 0.002, *** *p* < 0.001 using Friedman’s test.

Intragroup comparisons of all other periodontal parameters, namely PI, BOP, and CAL, showed significant improvements in all groups (Figure 2). However, intergroup comparisons indicated no significant changes between control and adjunctive interventions except for BOP, in which control sites exhibited significantly higher BOP scores than MB and TBO sites after 3 months (Figure 3).

## 4. Discussion

Periodontitis is one of the most frequent and widespread chronic inflammatory diseases affecting human beings [24]. The dysbiotic dental biofilm, particularly its Gram-negative bacteria such as *Porphyromonas gingivalis* and *Prevotella intermedia*, is the key etiological factor in the initiation and progression of PD [25]. SRD is the benchmark treatment method for PD. However, favorable outcomes of this treatment are not always ensured, and diverse responses could be observed between patients and even periodontal sites within the same patient. Different treatments were introduced as adjunct to RSD, such as antimicrobial therapy [5,7,9]. However, possible emergence of side effects and bacterial resistance in association with antibiotics highlighted PDT as a revolutionary solution for these issues [11,12]. Therefore, the present study aimed to examine the efficacy of MB and TBO as adjuncts to RSD in treatment of periodontitis. The results indicated superior clinical outcomes compared to the control, mainly for PPD and BOP, when PS (MB and TBO) were used as adjuncts to RSD.

The golden rule of periodontal therapy is to mechanically disturb subgingival microbiota to minimize the inflammatory events and restore periodontal tissues to a healthy state [26]. Conventionally, manual instrumentation using a range of specially designed curettes is the most followed approach for RSD. However, cumulative evidence suggests that ultrasonic-based devices and manual instrumentation are equally effective in treating periodontal pockets [18]. Additionally, root surfaces demonstrated rougher surfaces, more gouges, and thicker smear layers when curettes were used as compared to ultrasonic scalers [27,28].

PDT was selected due to two distinct advantages: Firstly, the PS is loaded directly into the periodontal pocket and can be activated by a laser or light emitting diode that is directed into the pocket, thus minimizing damage to neighboring host tissues [29]. Secondly, PDT is activated by light and results in a redox reaction in a relatively short period of time; therefore, bacterial resistance can be avoided [30]. The antimicrobial effect of PS is mainly derived from the release of reactive oxygen species which are responsible for damaging vital bacterial components such as proteins, lipids, and carbohydrates, resulting in the death of bacterial cells [31]. Additionally, MB was found to promote healing by inducing apoptosis of macrophages through the mitochondrial caspase pathway, reducing inflammation and bone resorption in a periodontitis rat model [32]. Both MB and TBO interact with lipopolysaccharides (LPS) that are present in the cell membrane of the Gram-negative bacteria and cause bacterial destruction when activated via release of free oxygen radicals [33]. However, TBO showed superior action against red complex bacteria such as *P. gingivalis* but exhibited a lower effect against other periodontal pathogens such as *Aggregatibacter actinomycetemcomitans* and *F. nucleatum* [34]. This could explain why better improvement of deep periodontal pockets, dominated by red complex pathogens, was associated with TBO more than MB.

The outcomes of this randomized clinical trial were judged based on closure of periodontal pockets 3 months after terminating active periodontal therapy, which is a clinically relevant measurement for failure/success of RSD [23,35]. The results of this study indicated both MB and TBO exhibited statistically significant reduction in the mean PPD as compared to the control group. These results suggested that treatment combining PDT with RSD provides additional benefit in reducing PPD and this improvement is commensurate with the results of previous studies [36,37,38,39] where PPD reduction was reported to be statistically significant when PDT was combined with RSD. Additionally, TBO was shown to be more effective in treating deep pockets (>6 mm) than MB. Meanwhile, MB demonstrated better clinical efficacy in moderate pockets (4–6 mm). However, TBO has shown a higher antibacterial effect on the isolated bacterial strains than MB [32]. The presence of more pathogenic bacteria might therefore explain the better improvement of deeper pockets by TBO. Nonetheless, both PS have been shown to be active against the pathogens in their environment, which grow in complex polymicrobial communities [40].

The findings of this study revealed that the use of two sessions of PDT application as an adjunct to RSD achieved statistically significant improvements in BOP% when compared to RSD alone. This can be explained by the fact that bacterial LPS uptake PS and, when bound to TLR4, stimulate and activate macrophages. This LPS–TLR4–PS complex is engulfed by macrophages as well [32]. Therefore, when PS-mediated bacterial death occurs, it will consequently cause macrophage destruction as well; hence, the PDT halts or minimizes the inflammation. Indeed, reduction of BOP scores is an essential parameter for successful periodontal therapy. BOP was suggested as a predictor of future clinical attachment loss [41] and Lang et al. [42] proposed that the absence of BOP can be used as a criterion for stability. Similar to the current study, improvements in BOP were reported by other studies when adjunctive PDT was used with RSD as compared to SRD alone [39,43,44]. These findings are consistent with those of Lang and coauthors who emphasized that decrease in BOP score coincides with diminution in periodontal inflammation [42].

The current study showed that PDT resulted in a non-statistically significant reduction in the PI% in comparison to controls. Undoubtedly, plaque removal is essential to control inflammation and prevent periodontal destruction. However, while PI can be seen as a tool to monitor patient compliance, it cannot be considered as a reliable measure to determine the effect of PDT.

Generally, the findings of the current study coincided with other studies that reported statistically significant improvements in BOP and CAL when using PDT as an adjunct to RSD [38,45,46]. However, other studies did not show any improvement in using MB- and TBO-mediated PDT in the treatment of PD [47,48,49]. These discrepancies in the results among the different studies could be associated with the difference in settings, such as the concentration of PS, period of retention of the PS within the tissue, time for biological response, pH of the environment (tissue/tooth interface), the presence of exudates and gingival fluid, and mode of PS application (irrigation, slow-release gel), in addition to the use of different methodologies, such as different optical fiber diameters and different irradiation parameters and wavelength settings [17]. In fact, the aforementioned discrepancies are the reasons for the lack of consensus on using PDT in routine periodontal therapy [18], indicating the need for standardizing the PDT treatment protocols.

This work presented the outcomes of PDT on clinical parameters only, and future studies should further confirm these results through microbiological and immunological assays. Indeed, the inclusion of molar teeth with furcation involvement and evaluating the outcomes over a longer period of time are recommended. However, this study showed promising improvement in clinical parameters that should be carefully interpreted before being translated into dental practice.

## 5. Conclusions

The findings of the current trial suggested that combining MB- and TBO-mediated PDT with RSD enhanced periodontal pocket closure and reduced signs of inflammation in comparison to the standard treatment with RSD alone. Interestingly, the results indicated that MB was more efficacious in moderately deep pockets, whereas TBO showed better efficacy in treating deeper pockets. This could provide a basis for recommending specific PS in clinical practice, depending on the severity of periodontitis, which should be confirmed by conducting further trials.

## Figures and Tables

**Table 1:** Demographic and clinical variables for study groups.

**Sex (*n*, %)**		**Age (Mean ± SD)**	
Male	9, 50%	44.0 ± 9.1	
Female	9, 50%	42.9 ± 12.9	
Total	18, 100%	43.4 ± 10.9	
**Sites (*n*, %)**	**All sites**	**4 to 6 mm**	**>6 mm**
Control	102, 30.7%	73, 32.7%	29, 26.6%
MB	124, 37.3%	77, 34.5%	33, 30.3%
TBO	106, 31.9%	73, 32.7%	47, 43.1%
Total	332, 100%	223, 100%	109, 100%

MB: methylene blue, TBO: toluidine blue O.

**Table 2:** Inter- and intra-group comparisons of probing pocket depth reduction.

PPD	Control ^†^	MB ^†^	TBO ^†^
Baseline	5.8 ± 1.7	5.9 ± 1.6	6.2 ± 1.4
3 months	4.0 ± 1.2	3.1 ± 1.0	3.4 ± 1.1
*p* value *	<0.001	<0.001	<0.001
Effect size ^‡^		0.7	0.5
ΔPPD _(3-month baseline)_	−1.8 ± 0.8	−2.8 ± 0.9 **	−2.8 ± 1.1 **
4 to 6 mm			
Baseline	4.8 ± 0.6	5.0 ± 0.7	5.1 ± 0.5
3 months	3.3 ± 0.9	2.7 ± 0.9	2.9 ± 0.8
*p* value *	<0.001	<0.001	<0.001
Effect size ^‡^		0.3	0.6
ΔPPD _(3-month baseline)_	−1.5 ± 0.5	−2.3 ± 0.8 **	−2.1 ± 0.7 **
>6 mm			
Baseline	8.1 ± 1.1	7.8 ± 0.8	8.0 ± 1.0
3 months	5.4 ± 1.2	4.2 ± 0.8	4.4 ± 0.8
*p* value *	<0.001	<0.001	<0.001
Effect size ^‡^		0.9	0.7
ΔPPD _(3-month-Baseline)_	−2.6 ± 0.7	−3.5 ± 1.0 **	−3.6 ± 1.1 **

MB: methylene blue, TBO: toluidine blue O, PPD: probing pocket depth. ^†^ Mean ± SD. ^‡^ Effect size by Cohen’s d formula. * Significant difference at *p* < 0.05 by Wilcoxon signed-rank test. ** Significant difference from control group at *p* < 0.05 by Kruskal–Wallis test.

**Table 3:** Distribution and inferential analysis of periodontal pockets pre- and post-treatment for all study groups.

Pockets Closure	Endpoint Achieved ^†^		*p* Value *	Odds Ratio	95% CI
PPD ^§^	Yes	No			
Control	60, 18.1%	42, 12.7%		1	
MB	99, 29.8%	25, 7.5%	<0.001	3.422	1.950 to 5.669
TBO	88, 26.5%	18, 5.4%	<0.001	2.772	1.704 to 4.451
Total	247, 74.4%	85, 25.6%			
4 to 6 mm ^§^					
Control	44, 19.7%	29, 13.0%		1	
MB	61, 26.5%	12, 5.4%	0.003	3.350	1.722 to 6.618
TBO	59, 27.4%	18, 8.1%	0.04	2.160	1.222 to 3.948
Total	164, 73.5%	59, 26.5%			
>6 mm ^§^					
Control	16, 14.7%	13, 11.9%		1	
MB	27, 24.8%	6, 5.5%	0.03	3.656	1.395 to 9.198
TBO	40, 36.7%	7, 6.4%	0.007	4.643	1.929 to 10.270
Total	83, 76.1%	26, 23.9%			

MB: methylene blue, TBO: toluidine blue O, PPD: probing pocket depth, CI: confidence interval. ^§^ Frequency, percent. ^†^ Endpoint: reduction of mean PPD by 1.29 mm for 4–6 mm pockets and 2.16 mm for pockets > 6 mm after 3 months. * Significant difference at *p* < 0.05 by Chi-square test.

## Data Availability

The raw data supporting the conclusions of this article will be made available by the authors on request.
